# Tetra­methyl­ammonium hemi(terephthalate) dihydrate

**DOI:** 10.1107/S1600536811023312

**Published:** 2011-06-22

**Authors:** Yunxia Yang, Laipeng Shi

**Affiliations:** a Key Laboratory of Polymer Materials of Gansu Province, Ministry of Education, College of Chemistry and Chemical Engineering, Northwest Normal University, Lanzhou 730070, Gansu, People’s Republic of China

## Abstract

In the title compound, (CH_3_)_4_N^+^·0.5C_8_H_4_O_4_^2−^·2H_2_O, the complete terephthalate dianion is completed by twofold symmetry and has a dihedral angle of 23.5 (2)° between the carboxyl­ate group and its parent ring. Two independent water mol­ecules serve as both donors and acceptor in the construction of undulating hydrogen-bonded host layers with various O—H⋯O contacts ocurring between the anion and two water mol­ecules. At the same time, the tetra­methyl­ammonium cations, as the sphere-like guest species, are arranged in two rows between neighboring host layers, with an approximate inter­layer distance of 7.36 Å, forming a sandwich-like crystal structure.

## Related literature

Biphenyl-4,4′-dicarb­oxy­lic acid can be used as a host mol­ecule in the construction of different host–guest crystal structures with various cations such as tetra­ethyl­ammonium and choline ions, see: Furey *et al.* (1996 ▶); Xu *et al.* (2002 ▶).
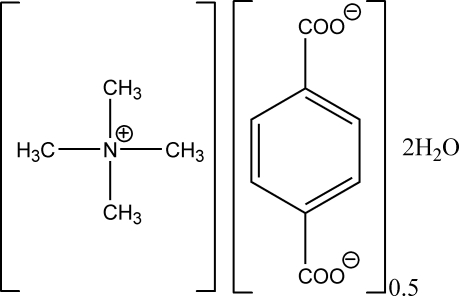


## Experimental

### 

#### Crystal data

C_4_H_12_N^+^·0.5C_8_H_4_O_4_^2−^·2H_2_O
*M* *_r_* = 192.23
Monoclinic, 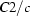

*a* = 22.0950 (4) Å
*b* = 11.2922 (2) Å
*c* = 9.1101 (1) Å
β = 109.613 (1)°
*V* = 2141.10 (6) Å^3^
*Z* = 8
Mo *K*α radiation
μ = 0.09 mm^−1^
*T* = 296 K
0.23 × 0.16 × 0.10 mm

#### Data collection

Bruker APEXII CCD area-detector diffractometer
Absorption correction: multi-scan (*SADABS*; Bruker, 2009 ▶) *T*_min_ = 0.979, *T*_max_ = 0.991
6025 measured reflections
2227 independent reflections
1771 reflections with *I* > 2σ(*I*)
*R*_int_ = 0.015

#### Refinement

*R*[*F* ^2^ > 2σ(*F* ^2^)] = 0.047
*wR*(*F* ^2^) = 0.140
*S* = 1.03
2227 reflections
118 parameters
2 restraints
H-atom parameters constrained
Δρ_max_ = 0.19 e Å^−3^
Δρ_min_ = −0.24 e Å^−3^

### 

Data collection: *APEX2* (Bruker, 2009 ▶); cell refinement: *SAINT* (Bruker, 2009 ▶); data reduction: *SAINT*; program(s) used to solve structure: *SHELXS97* (Sheldrick, 2008 ▶); program(s) used to refine structure: *SHELXL97* (Sheldrick, 2008 ▶); molecular graphics: *SHELXTL* (Sheldrick, 2008 ▶); software used to prepare material for publication: *SHELXL97* and *publCIF* (Westrip, 2010 ▶).

## Supplementary Material

Crystal structure: contains datablock(s) I, global. DOI: 10.1107/S1600536811023312/hg5050sup1.cif

Structure factors: contains datablock(s) I. DOI: 10.1107/S1600536811023312/hg5050Isup2.hkl

Supplementary material file. DOI: 10.1107/S1600536811023312/hg5050Isup3.cml

Additional supplementary materials:  crystallographic information; 3D view; checkCIF report

## Figures and Tables

**Table 1:** Hydrogen-bond geometry (Å, °)

*D*—H⋯*A*	*D*—H	H⋯*A*	*D*⋯*A*	*D*—H⋯*A*
O1*W*—H1*WA*⋯O2	0.87	1.87	2.7262 (17)	168
O1*W*—H1*WB*⋯O1*W*^i^	0.84	2.41	2.812 (2)	110
O2*W*—H2*WA*⋯O1^ii^	0.86	1.89	2.7291 (15)	164
O2*W*—H2*WB*⋯O1^iii^	0.86	1.96	2.7999 (18)	165

Symmetry codes: (i) 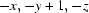; (ii) 
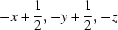
; (iii) 
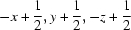
.

**Fig. 1.**
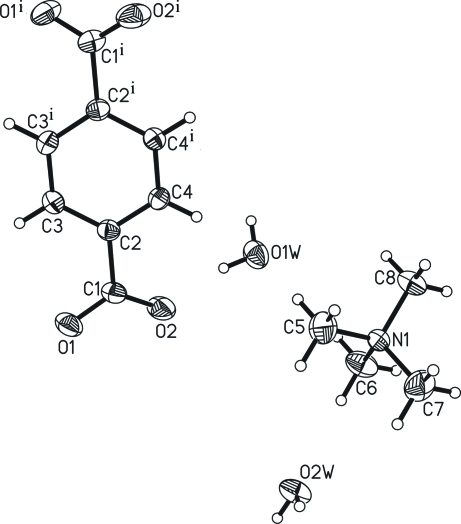
Thermal ellipsoid plot of the title compound at the 30% probability level; hydrogen atoms are drawn as spheres of arbitrary radius [Symmetry code: (i) -x, y, -z + 1/2.].

**Fig. 2.**
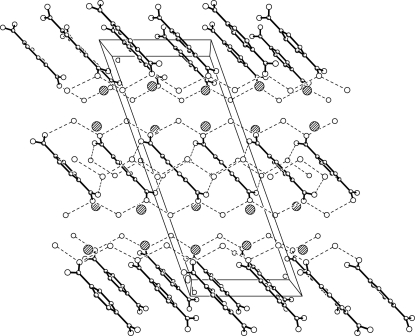
Packing diagram of the title compound; all hydrogen atoms are omitted for clarity and the cations are represented with the hatched spheres.

**Fig. 3.**
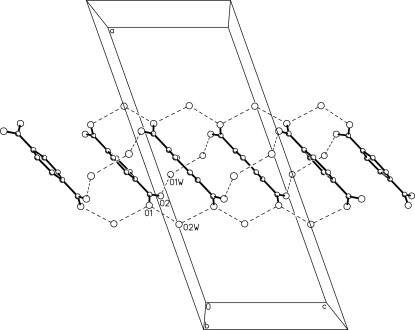
Hydrogen bond pattern in the host layer of the title compound; all hydrogen atoms are omitted for clarity.

## supplementary crystallographic information

### Comment

Biphenyl-4,4'-dicarboxylic acid can be used as host molecule to construct different host–guest crystal structures with various cations such as tetraethylammonium and choline ions (Furey *et al.*, 1996; Xu *et al.*, 2002). In this structure, there is half a terephthalate anion disposed at the twofold axis, two water molecules, and one tetramethylammonium cation at general positions in the asymmetric unit. From the packing diagram (Fig. 2), it can be observed that terephthalate anion and two water molecules form hydrogen-bonded host layers along the *b* axis with the help of four various O—H···O hydrogen bonds between the anion and these two water molecules. The guest cations are doubly contained between the layers with an interlayer distance of *a*/3≈7.36 Å. Obviously, two independent water molecules, as the complementary host molecules, play a significant linking role in constructing the hydrogen-bonded host layer by generating four O—H···O hydrogen bonds (Fig. 3).

### Experimental

Biphenyl-4,4'-dicarboxylic acid (0.042 g, 0.25 mmol) was dissolved in a water-ethanol (1:2 *v*/*v*) mixture and tetramethylammonium hydroxide was added to neutralize the acid. Colorless block crystals formed after several days.

### Refinement

All hydrogen atoms bonded to carbon were introduced to idealized positions and allowed to ride on their parent atoms. Hydrogen atoms bonded to oxygen were located in difference Fourier syntheses with O—H distance of 0.86 Å.

### Crystal data

**Table:**

C_4_H_12_N^+^·0.5C_8_H_4_O_4_^2^^−^·2H_2_O	*F*(000) = 840
*M**_r_* = 192.23	*D*_x_ = 1.193 Mg m^−^^3^
Monoclinic, *C*2/*c*	Mo *K*α radiation, λ = 0.71073 Å
Hall symbol: -C 2yc	Cell parameters from 2337 reflections
*a* = 22.0950 (4) Å	θ = 2.9–26.4°
*b* = 11.2922 (2) Å	µ = 0.09 mm^−^^1^
*c* = 9.1101 (1) Å	*T* = 296 K
β = 109.613 (1)°	Block, colorless
*V* = 2141.10 (6) Å^3^	0.23 × 0.16 × 0.10 mm
*Z* = 8	

### Data collection

**Table:**

Bruker APEXII CCD area-detector diffractometer	2227 independent reflections
Radiation source: fine-focus sealed tube	1771 reflections with *I* > 2σ(*I*)
graphite	*R*_int_ = 0.015
φ and ω scans	θ_max_ = 26.6°, θ_min_ = 2.1°
Absorption correction: multi-scan (*SADABS*; Bruker, 2009)	*h* = −15→27
*T*_min_ = 0.979, *T*_max_ = 0.991	*k* = −12→14
6025 measured reflections	*l* = −11→11

### Refinement

**Table:**

Refinement on *F*^2^	Primary atom site location: structure-invariant direct methods
Least-squares matrix: full	Secondary atom site location: difference Fourier map
*R*[*F*^2^ > 2σ(*F*^2^)] = 0.047	Hydrogen site location: inferred from neighbouring sites
*wR*(*F*^2^) = 0.140	H-atom parameters constrained
*S* = 1.03	*w* = 1/[σ^2^(*F*_o_^2^) + (0.0762*P*)^2^ + 0.7137*P*] where *P* = (*F*_o_^2^ + 2*F*_c_^2^)/3
2227 reflections	(Δ/σ)_max_ < 0.001
118 parameters	Δρ_max_ = 0.19 e Å^−^^3^
2 restraints	Δρ_min_ = −0.24 e Å^−^^3^

### Special details

**Table:**

Geometry. All e.s.d.'s (except the e.s.d. in the dihedral angle between two l.s. planes) are estimated using the full covariance matrix. The cell e.s.d.'s are taken into account individually in the estimation of e.s.d.'s in distances, angles and torsion angles; correlations between e.s.d.'s in cell parameters are only used when they are defined by crystal symmetry. An approximate (isotropic) treatment of cell e.s.d.'s is used for estimating e.s.d.'s involving l.s. planes.
Refinement. Refinement of *F*^2^ against ALL reflections. The weighted *R*-factor *wR* and goodness of fit *S* are based on *F*^2^, conventional *R*-factors *R* are based on *F*, with *F* set to zero for negative *F*^2^. The threshold expression of *F*^2^ > σ(*F*^2^) is used only for calculating *R*-factors(gt) *etc*. and is not relevant to the choice of reflections for refinement. *R*-factors based on *F*^2^ are statistically about twice as large as those based on *F*, and *R*- factors based on ALL data will be even larger.

### Fractional atomic coordinates and isotropic or equivalent isotropic displacement parameters (Å^2^)

**Table:**

	*x*	*y*	*z*	*U*_iso_*/*U*_eq_	
O1	0.13625 (6)	0.07836 (11)	0.13458 (15)	0.0647 (4)	
C1	0.10126 (7)	0.16860 (14)	0.11158 (17)	0.0484 (4)	
N1	0.16707 (5)	0.69678 (10)	0.18126 (13)	0.0424 (3)	
O1W	0.03390 (7)	0.44906 (11)	−0.09000 (15)	0.0711 (4)	
H1WA	0.0527	0.3822	−0.0561	0.107*	
H1WB	−0.0051	0.4402	−0.1035	0.107*	
O2	0.10772 (6)	0.25714 (12)	0.03731 (16)	0.0753 (4)	
C2	0.04837 (6)	0.16903 (12)	0.18277 (15)	0.0414 (3)	
O2W	0.30308 (6)	0.52166 (12)	0.05088 (15)	0.0716 (4)	
H2WA	0.3259	0.4825	0.0078	0.107*	
H2WB	0.3274	0.5319	0.1455	0.107*	
C3	0.02429 (8)	0.06406 (14)	0.2185 (2)	0.0593 (4)	
H3A	0.0411	−0.0076	0.1997	0.071*	
C4	0.02375 (7)	0.27422 (12)	0.21607 (15)	0.0399 (3)	
H4A	0.0392	0.3458	0.1927	0.048*	
C5	0.16656 (11)	0.58985 (19)	0.2752 (3)	0.0846 (6)	
H5A	0.2052	0.5452	0.2905	0.127*	
H5B	0.1299	0.5419	0.2214	0.127*	
H5C	0.1642	0.6132	0.3745	0.127*	
C6	0.17003 (10)	0.66356 (19)	0.0258 (2)	0.0727 (6)	
H6A	0.2087	0.6198	0.0389	0.109*	
H6B	0.1698	0.7339	−0.0335	0.109*	
H6C	0.1335	0.6155	−0.0283	0.109*	
C7	0.22409 (10)	0.7706 (2)	0.2642 (3)	0.0810 (6)	
H7A	0.2626	0.7260	0.2777	0.121*	
H7B	0.2223	0.7932	0.3642	0.121*	
H7C	0.2242	0.8404	0.2040	0.121*	
C8	0.10738 (8)	0.76601 (17)	0.1586 (2)	0.0628 (5)	
H8A	0.1051	0.7884	0.2583	0.094*	
H8B	0.0707	0.7185	0.1041	0.094*	
H8C	0.1078	0.8358	0.0989	0.094*	

### Atomic displacement parameters (Å^2^)

**Table:**

	*U*^11^	*U*^22^	*U*^33^	*U*^12^	*U*^13^	*U*^23^
O1	0.0632 (7)	0.0710 (8)	0.0713 (8)	0.0227 (6)	0.0374 (6)	0.0106 (6)
C1	0.0430 (8)	0.0600 (9)	0.0446 (8)	0.0082 (7)	0.0177 (6)	0.0054 (7)
N1	0.0390 (6)	0.0414 (6)	0.0456 (6)	0.0048 (5)	0.0126 (5)	0.0025 (5)
O1W	0.0854 (9)	0.0637 (8)	0.0681 (8)	0.0174 (7)	0.0311 (7)	0.0150 (6)
O2	0.0725 (8)	0.0844 (9)	0.0881 (9)	0.0251 (7)	0.0522 (7)	0.0361 (7)
C2	0.0374 (7)	0.0464 (8)	0.0395 (7)	0.0025 (6)	0.0118 (6)	0.0016 (6)
O2W	0.0567 (7)	0.0910 (9)	0.0671 (8)	0.0201 (7)	0.0206 (6)	−0.0087 (7)
C3	0.0581 (9)	0.0401 (8)	0.0893 (12)	0.0052 (7)	0.0375 (9)	−0.0021 (8)
C4	0.0432 (7)	0.0410 (7)	0.0355 (6)	−0.0016 (5)	0.0134 (6)	0.0027 (5)
C5	0.0846 (14)	0.0652 (12)	0.1042 (16)	0.0117 (10)	0.0319 (12)	0.0371 (11)
C6	0.0670 (11)	0.0936 (14)	0.0606 (10)	0.0172 (10)	0.0255 (9)	−0.0115 (10)
C7	0.0596 (11)	0.0790 (13)	0.0844 (14)	−0.0132 (10)	−0.0021 (10)	−0.0082 (11)
C8	0.0541 (10)	0.0708 (11)	0.0661 (10)	0.0205 (8)	0.0235 (8)	0.0041 (8)

### Geometric parameters (Å, °)

**Table:**

O1—C1	1.2534 (18)	C4—C4^i^	1.385 (3)
C1—O2	1.2420 (19)	C4—H4A	0.9300
C1—C2	1.5151 (19)	C5—H5A	0.9600
N1—C5	1.482 (2)	C5—H5B	0.9600
N1—C8	1.4867 (18)	C5—H5C	0.9600
N1—C6	1.487 (2)	C6—H6A	0.9600
N1—C7	1.488 (2)	C6—H6B	0.9600
O1W—H1WA	0.8668	C6—H6C	0.9600
O1W—H1WB	0.8351	C7—H7A	0.9600
C2—C4	1.3818 (19)	C7—H7B	0.9600
C2—C3	1.382 (2)	C7—H7C	0.9600
O2W—H2WA	0.8580	C8—H8A	0.9600
O2W—H2WB	0.8570	C8—H8B	0.9600
C3—C3^i^	1.377 (3)	C8—H8C	0.9600
C3—H3A	0.9300		
			
O2—C1—O1	124.64 (14)	H5A—C5—H5B	109.5
O2—C1—C2	118.47 (13)	N1—C5—H5C	109.5
O1—C1—C2	116.88 (13)	H5A—C5—H5C	109.5
C5—N1—C8	109.28 (14)	H5B—C5—H5C	109.5
C5—N1—C6	110.81 (16)	N1—C6—H6A	109.5
C8—N1—C6	108.73 (13)	N1—C6—H6B	109.5
C5—N1—C7	109.44 (15)	H6A—C6—H6B	109.5
C8—N1—C7	109.64 (14)	N1—C6—H6C	109.5
C6—N1—C7	108.92 (15)	H6A—C6—H6C	109.5
H1WA—O1W—H1WB	107.2	H6B—C6—H6C	109.5
C4—C2—C3	118.33 (13)	N1—C7—H7A	109.5
C4—C2—C1	120.92 (12)	N1—C7—H7B	109.5
C3—C2—C1	120.75 (13)	H7A—C7—H7B	109.5
H2WA—O2W—H2WB	105.3	N1—C7—H7C	109.5
C3^i^—C3—C2	120.92 (8)	H7A—C7—H7C	109.5
C3^i^—C3—H3A	119.5	H7B—C7—H7C	109.5
C2—C3—H3A	119.5	N1—C8—H8A	109.5
C2—C4—C4^i^	120.73 (8)	N1—C8—H8B	109.5
C2—C4—H4A	119.6	H8A—C8—H8B	109.5
C4^i^—C4—H4A	119.6	N1—C8—H8C	109.5
N1—C5—H5A	109.5	H8A—C8—H8C	109.5
N1—C5—H5B	109.5	H8B—C8—H8C	109.5

Symmetry codes: (i) −*x*, *y*, −*z*+1/2.

### Hydrogen-bond geometry (Å, °)

**Table:**

*D*—H···*A*	*D*—H	H···*A*	*D*···*A*	*D*—H···*A*
O1W—H1WA···O2	0.87	1.87	2.7262 (17)	168
O1W—H1WB···O1W^ii^	0.84	2.41	2.812 (2)	110
O2W—H2WA···O1^iii^	0.86	1.89	2.7291 (15)	164
O2W—H2WB···O1^iv^	0.86	1.96	2.7999 (18)	165

Symmetry codes: (ii) −*x*, −*y*+1, −*z*; (iii) −*x*+1/2, −*y*+1/2, −*z*; (iv) −*x*+1/2, *y*+1/2, −*z*+1/2.
